# Prediction of radio-responsiveness with immune-profiling in patients with rectal cancer

**DOI:** 10.18632/oncotarget.19558

**Published:** 2017-07-25

**Authors:** In Ja Park, Soyeon An, Sang-Yeob Kim, Hye Min Lim, Seung-Mo Hong, Mi-Ju Kim, Yun Jae Kim, Chang Sik Yu

**Affiliations:** ^1^ Department of Colon and Rectal Surgery, University of Ulsan College of Medicine and Asan Medical Center, Seoul, Korea; ^2^ Department of Pathology, University of Ulsan College of Medicine and Asan Medical Center, Seoul, Korea; ^3^ Department of Convergence Medicine, University of Ulsan College of Medicine and Asan Medical Center, Seoul, Korea; ^4^ Asan Institute for life sciences, Asan Medical Center, Seoul, Korea; ^5^ Department of Pathology, Incheon St. Mary's Hospital, College of Medicine, The Catholic University of Korea, Incheon, Korea

**Keywords:** rectal cancer, preoperative chemoradiotherapy, responsiveness, immune infiltrates

## Abstract

We evaluate whether the tumor immune infiltrate (TIL) could be used for prediction of responsiveness to preoperative chemoradiotherapy (PCRT) in rectal cancers. Using formalin-fixed paraffin-embedded slides of pretreatment biopsies, co-stain for CD4, CD8, CD274 (PD-L1), FOXP3, cytokeratin, and DAPI was performed with *Opal multi staining kit* (Perkin-Elmer, Waltham, MA). Multispectral imaging and digital analysis to visualize and quantify specific immune infiltrates were performed using the *Vectra imaging system* (Perkin-Elmer). The density (number of cells per mm^2^) and proportion of total TILs and specific cell types in the stroma were calculated by inForm™ 2.2.1 software (Perkin-Elmer). The density and proportion of total TILs and specific cell types in the stroma were calculated by inForm™ 2.2.1 software (Perkin-Elmer, Waltham, MA). Patients were classified as group with total regression (TR, *n* = 25) and group with residual disease (near total, moderate, and minimal regression, RD, *n =* 50). The mean density of T cell infiltration and CD274 (PD-L1)+ lymphocyte were significantly higher in TR (*p* = 0.005, *p* = 0.001). The proportion of CD4+ lymphocyte (p=0.042) and CD274 (PD-L1)+ lymphocyte (*p* = 0.002) were different between 2 groups. The TR group has lower CD4+ and higher CD274 (PD-L1)+ proportions than RD group. The ratio among CD4+, CD8+, CD274 (PD-L1)+, FOXP3+ T cell was different between groups. TR group showed lower CD4/ CD274 (PD-L1) (*p* = 0.007), CD8/ CD274 (PD-L1) (*p* = 0.02), and FOXP3/ CD274 (PD-L1) (*p* = 0.003) ratio than RD group. The determination of the immune infiltrate in biopsies before treatment could be a valuable information for the prediction of responsiveness to PCRT.

## INTRODUCTION

The preoperative chemoradiotherapy (PCRT) was reported to decrease local recurrence, induce tumor down-staging, and allow sphincter preservation [1–3] in patients with locally advanced rectal cancer. In addition, 12–30% of the patients who received PCRT showed total regression of primary tumor [1, 3, 4]. Patients with total regression to PCRT expected to have good oncologic outcomes [4, 5] and might even have a potential for organ-preserving strategy [4, 6]. On the contrary, some of the patients who did not show significant regression of tumor to PCRT resulted in exposure to in-effective treatment.

The ability to predict tumor responses before PCRT would significantly impact the selection of patients for PCRT as well as potentially modifying postoperative treatment plans. However, the clinical and radiological features were disappointing than was expected for predicting responsiveness [7–9] therefore, researches to search molecular predictors of rectal cancer response to PCRT have been great interest. Although extensive researches have been conducted, it is hard to identify reliable predictive marker because complex mechanisms were involved in resistance to radiation therapy [10–12]. In addition to genetic alteration, immune components also were reported to be associated with PCRT responsiveness as well as oncologic outcome in rectal cancer patients [13].

Accumulating evidence suggests that effector/cytotoxic CD3+ and CD8+ and memory CD45RO+ T cells play important roles in the anti-tumor immune response [13–15]. High levels of tumor infiltrating lymphocytes (TILs) have shown to be associated with improved oncologic outcomes of colorectal cancer patients. Currently, interest in immunotherapy is increasing based on understanding of association immune components and oncologic outcomes.

Evidences for immune component to radiation therapy effect [13, 16, 17] were suggested and response to PCRT would be influenced by immune status. Although the mechanism remains uncertain, we expect that PCRT is immune adjuvant acting through immune response [18]. Therefore, immune components have to be considered together with genetic feature of tumor to develop predictive marker for PCRT responsiveness.

In the present study, we evaluate difference of the immune-infiltrates profiling according to tumor responsiveness to PCRT and how to use immune-infiltrates profiling for prediction of radioresponsiveness in rectal cancer patients.

## RESULTS

### Patient characteristics

The analysis included 52 (69.3%) men, and the median age was 56 years (interquartile range, 50–65). Total regression group and residual disease group showed no difference in terms of age, gender, tumor distance from anal verge, pre-PCRT CEA, and cT stage. Of the 75 patients, ypT3 which was 23 (30.7%) was the most frequently reported on pathologic examination. Microsatellite status was evaluated in 56 patients and 2 showed microsatellite instability. Metastatic lymph node was identified in 13 patients (17.4%) on pathologic examination (Table 1). Using tumor regression grade (TRG) criteria, 25 (50%) showed near total regression, 11 (22%) moderate regression, and 14 (28%) minimal regression among residual disease group.

**Table 1:** Clinicopathological characteristics of the study patients

Variable	Total regression	Residual disease	*P*
**Age, median (IQR)**	59 (51–69)	54 (49–64)	0.129
**Gender**			0.859
Male	17 (68%)	35 (70%)	
Female	8 (32%)	15 (30%)	
**Distance from anal verge, cm, median (IQR)**	4 (3–6)	4(3–5)	0.719
**Pre-PCRT CEA, ng/dL, median (IQR)** Within normal range, ≤ 6 ng/mL Increased	2.2 (1.8–3.5) 21 (84%) 4 (16%)	3.5(1.6–7.3) 36 (72%) 14 (28%)	0.275 0.251
**Clinical T stage before PCRT** cT2 cT3 cT4	2 (8%) 20 (80%) 3 (12%)	3 (6%) 38 (76%) 9 (18%)	0.775
**ypT stage** ypT0 ypT1 ypT2 ypT3 ypT4	25 (100%) - - - -	0 8 (16%) 16 (32%) 23 (46%) 3 (6%)	
**ypN stage** ypN0 ypN1 ypN2	25 (100%) - -	37 (74%) 11 (22%) 2 (4%)	0.02

IQR, interquartile range; PCRT, preoperative chemoradiotherapy; CEA, carcinoembryonic antigen

### Difference in density of immune-infiltrates, proportion, and ratio according to tumor response group

**Figure 1:**
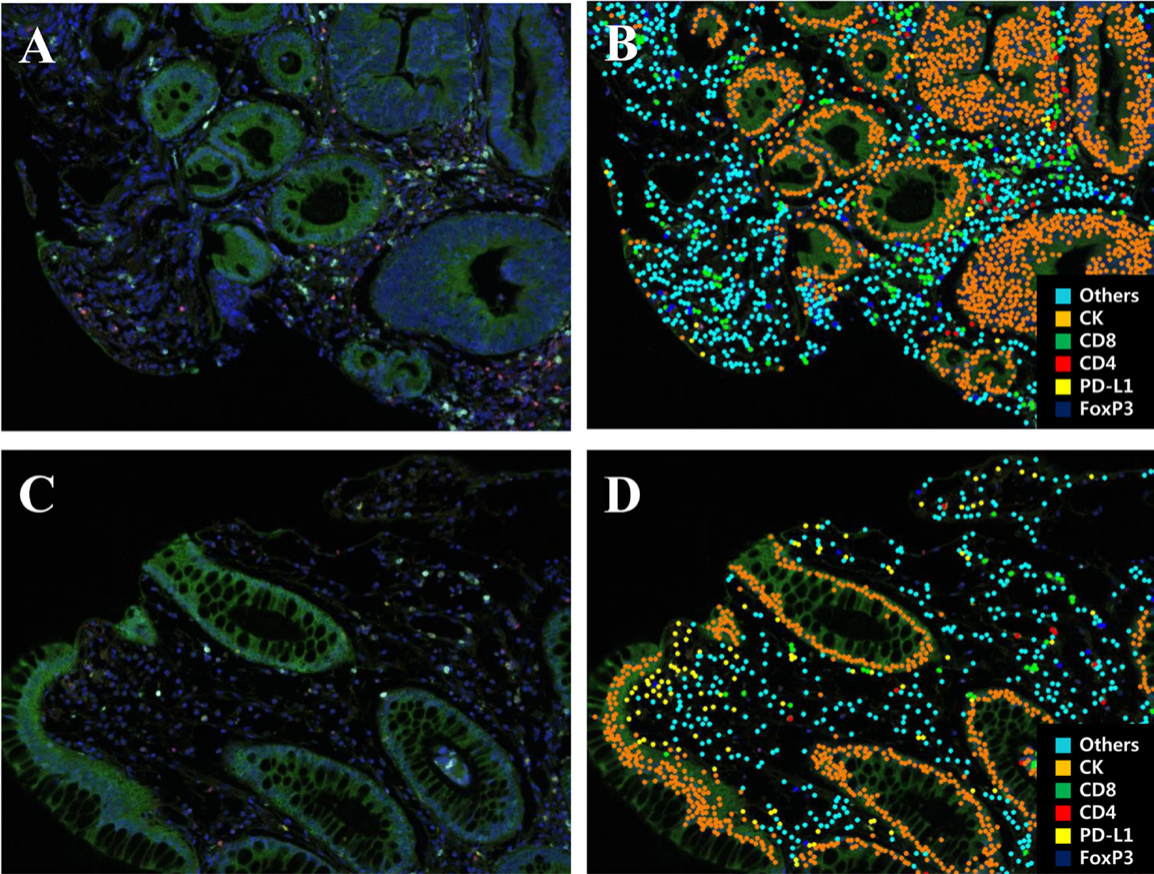
Representative multiplex IF images of pretreatment rectal cancer biopsy tissue (**A, C**) A spectral composite image created according to the spectral library for each fluorescent probe (Opal 690, Opal 650, Opal 620, Opal 570, Opal 520, and DAPI). (**B, D**) Cell phenotype map of rectal cancer biopsy tissue as a previously designated color (Blue, FoxP3; Yellow, PD-L1; Red, CD4; Green, CD8; Orange, cytokeratin; Sky blue, others).

M-IF assay for analysis of immune-infiltrates was performed in all 75 cases. Each section was sequentially stained with cytokeratin, FOXP3, CD274 (PD-L1), CD4, and CD8 in that order. Multispectral imaging successfully captured and quantified multiple immune cell types and CD274 (PD-L1) expression. After obtaining the whole scanned image, the tumor and the normal region were determined and the immune cells were counted (Figure 1). The number of evaluated block which was influenced by size of biopsy specimen was different by patients. In addition, the number of immune-infiltrates in the same patient was various or each evaluated block. Therefore, the density of immune-infiltrates was calculated using mean number of immune-infiltrates of all evaluated block. Total density of T-cell infiltrates was higher in total regression than in residual disease group (*p* = 0.005). The density of CD 4+, CD8+, and FOXP3+ T lymphocyte was not significantly different between tumor response groups. The density of CD274 (PD-L1)+ T lymphocyte was higher in TR than RD group (*p* = 0.001).

**Figure 2:**
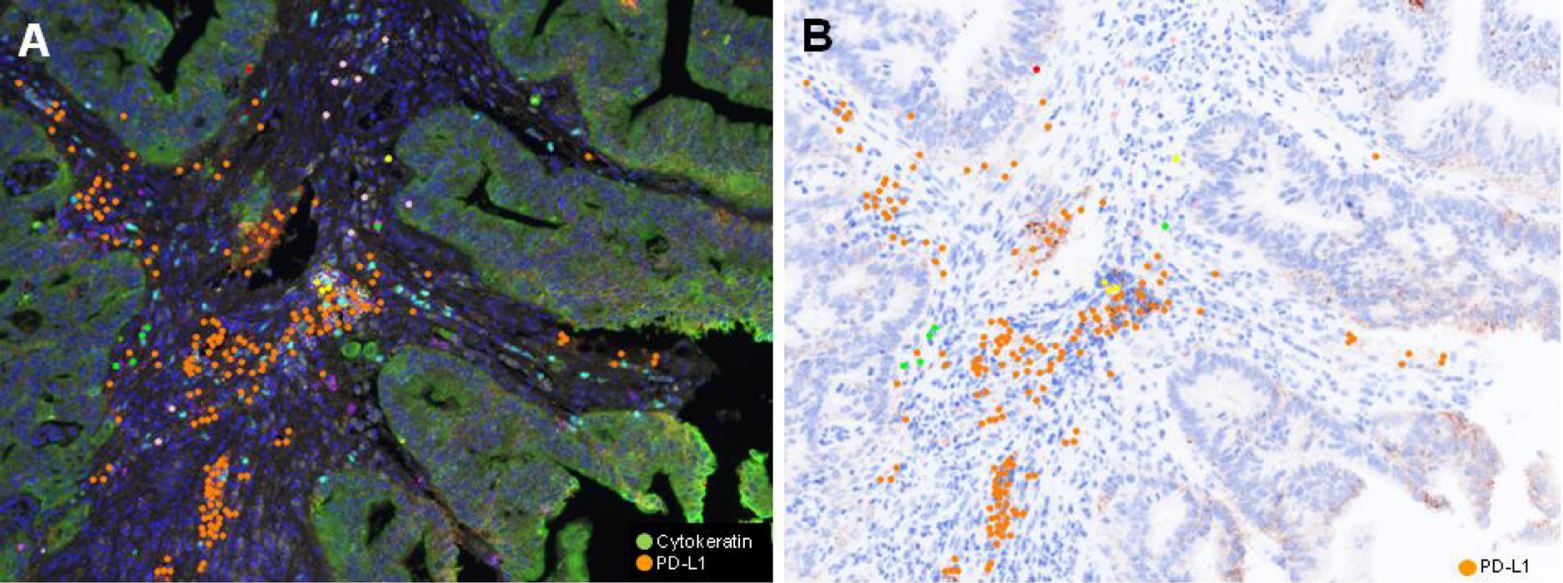
Representative (**A**) Multiplex IF image and (**B**) PD-L1 immunohistochemical inage of pretreatment rectal cancer biopsy of TR group (Case #12). A) Pseudocolor image. Cancer cells are green colored, while PD-L1 positive lymphoid cells are orange colored. PD-LI positive cells are in stroma, but not observe in cancer cells.

**Figure 3:**
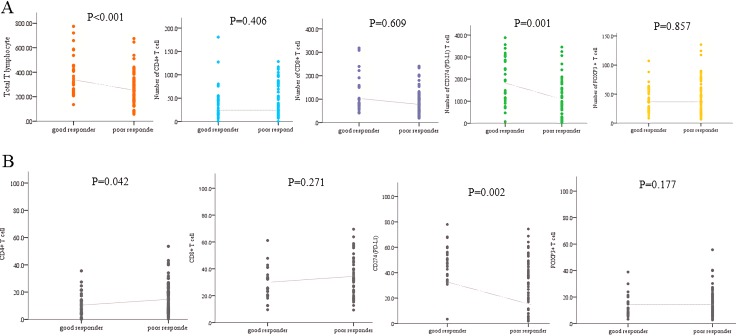
Differences in (**A**) density and (**B**) proportion of specific immune infiltrates between total regression and residual disease group.

The proportion of each type of immune infiltrates was compared. The proportion of CD4+ T cell was significantly higher in RD group (*p* = 0.042). However, proportion of CD274 (PD-L1)+ T cell was higher in TR group (*p* = 0.002, Figure 2). The proportion of CD 8+ and FOXP3+ T lymphocyte was higher in RD group, but those did not show statistical significance. The proportion of CD274 (PD-L1) showed inverse correlation with FOXP3+ (*p* = 0.005, r= 0.321), CD8+ (*p* < 0.001, *r* = 0.655) and CD4+ (*p* < 0.001, *p* = 0.519) T cell proportion (Figure 3).

### Predictability of immune-infiltrates profile and associated factors with total regression with primary tumor

**Figure 4:**
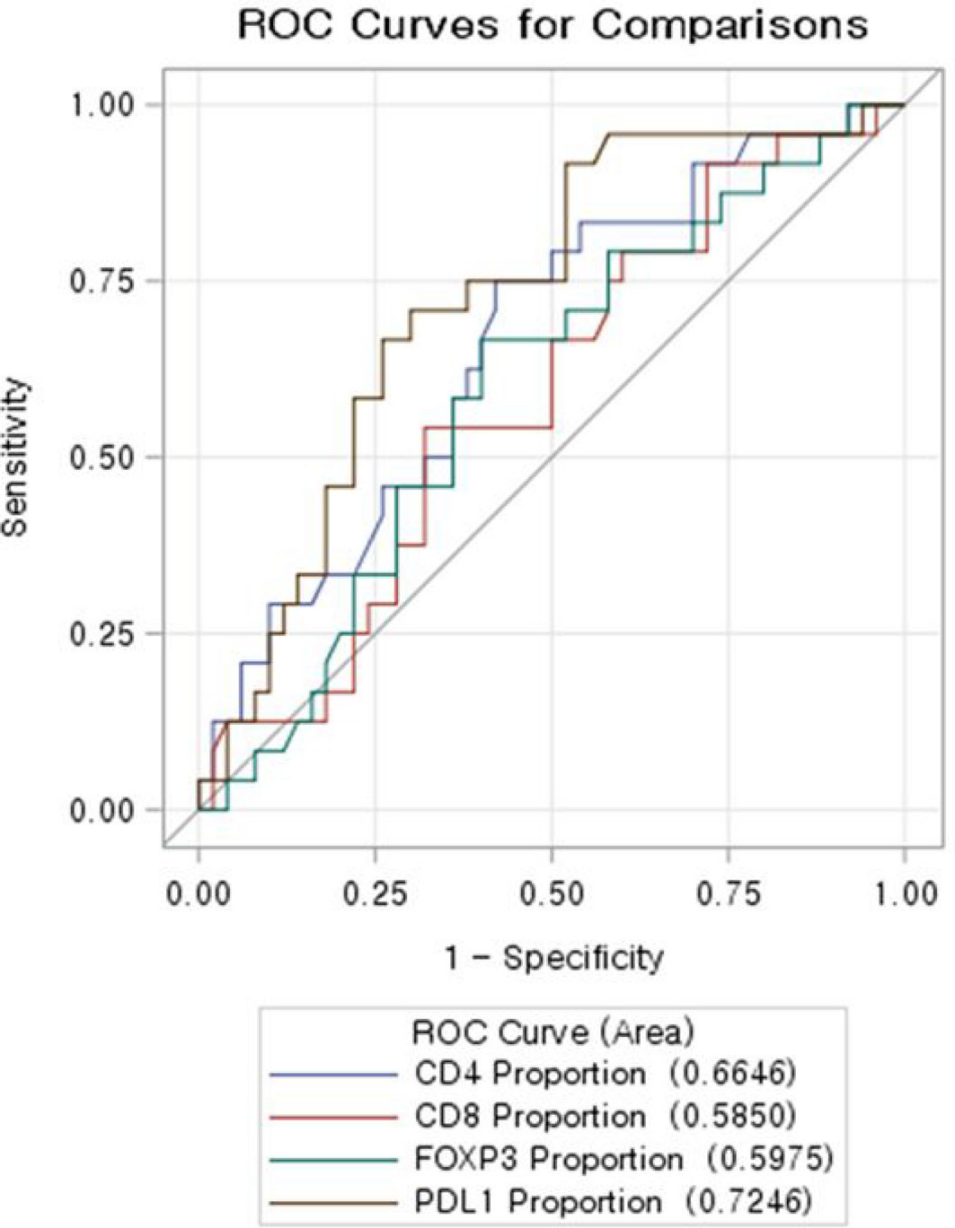
ROC curve evaluating predictability of total regression of immune-infiltrates profile CD 274(PD-L1) showed the highest AUC (area under curve).

Using ROC curve, CD274(PD-L1) proportion showed the highest predictability of total regression (Figure 4). ROC contrast estimation and testing results by row revealed the significant difference of AUC (area under curve) of CD274 (PD-L1) comparing CD4+, CD8+, and FOXP3 . Multivariate analysis was done to identify factors with total regression of primary tumor. CD 274(PD-L1) proportion was confirmed as independent associated factors of total regression when age, gender, location of tumor, pretreatment CEA, clinical T stage were adjusted (Table 2).

**Table 2:** Factors associated with total regression after preoperative chemoradiotherapy; multivariate analysis

Variable	Odd Ratio	95% confidence interval	*p*
Age	1.03	0.981–1.082	0.232
Gender Male Female	1 0.895	0.28–2.875	0.851
Pretreatment CEA Normal range Increased	1 1.954	0.472–8.098	0/356
cT stage cT2 cT3 cT4	1 1.11 2.11	0.105–11.803 0.386–11.511	0.372
CD274(PD-L1) proportion	1.046	1.012–1.081	0.007

## DISCUSSION

We found that specific immune infiltrates in pretreatment tissue of rectal cancer was different according to tumor response to preoperative chemoradiotherapy. Total density of T-cell infiltrates and CD274 (PD-L1)+ cell infiltrates were main discriminator between patients with total regression and residual disease after PCRT

There have been increasing reports regarding association between tumor infiltrating T-cells and prognosis of colorectal cancer patients [15–20] Tumor-infiltrating lymphocytes may also reflect specific molecular alterations associated with indolent tumor behavior. Tumor-induced immune suppression in cancer patients is a major issue that not only promotes tumor progression but also inhibits the efficiency of anti-cancer treatment [21, 22] Radiotherapy (RT) also involved in host immune effecter mechanisms that may contribute to the control and/or eradication of cancer [23, 24]. Therefore, the identification and inhibition of key drivers of immunosuppression of the tumor have the potential to improve patient outcome and increase treatment response.

We choose CD4, CD8, FOPX3, and CD274 (PD-L1) in the present study to assess radio-responsiveness. There have been reported many tumor infiltrating T-cells markers associated with colon cancer prognosis, but, we have to select limited number of markers because multiplex immunofluorescence study used in the present study could evaluated 6 markers simultaneously. Cytokeratin and DAPI had to be included to assess tumor and background. Therefore we could include only 4 markers to assess radio-responsiveness. We decided to use CD4 and CD8 to assess numerically cellular immunity. We select FOXP3 to evaluate immune tolerance. CD274 was included in marker because it could be used as a marker of immune-check point inhibitor.

In the present study, CD274 (PD-L1) expression was increased in total regression group (TR). Although CD8+ T-cell and Tregs were higher in residual disease group (RD), it did not show statistical significance contrary to published previous studies. Hypothetically, CD274 (PD-L1) was involved in immune-suppression and might be overexpressed in “worse prognostic group” and possibly resistant to anti-tumor therapy. Recently, CD274 (PD-L1) was interested as a promising immunotherapy target in cancer treatment. A CD274 (PD-L1), a major molecular regulator of tumor immune escape, inhibits T cell-mediated immune attack by binding to the PD-1 receptor on tumor-specific T cells [25, 26]. The colorectal tumors expressing CD274(PD-L1) was reported that associated with poorly prognostic factors such as poorly differentiation, BRAF mutation and ‘stem-like’ immunophenotype features [29]. Masugi et al. showed that CD274 expression level is inversely associated with the density of FOXP3+ lymphocytes in colorectal carcinoma tissue which reflect tumor CD274 expression would influence on regulatory T-cell [30]. They calculated FOXP3+cell density and analysis correlation with CD274 expression using immunohistochemistry (IHC).

In the present study, we calculate immune-infiltrates and evaluated the correlation between proportion of CD274(PD-L1) and other immune infiltrates. Because tissue amount was different among each patients, we evaluated proportion-based correlation. CD274(PD-L1) inversely correlated with FOXP3+, CD8+, and CD4+ T-cell proportion. Therefore, we can suggest that CD274(PD-L1) expression in tumor stroma would influence on immune reaction in the setting of preoperative chemoradiotherapy. Therefore, further studies which find mechanism between the immune checkpoint pathway and host immunity in radioresponsiveness of rectal cancer patients.

Emerging evidence suggests that the generation of antitumor immune responses might play an important role in the effectiveness of radiotherapy [27, 28]. Radiation increased the T cell recognition of irradiated tumor cells, making the cells vulnerable to cytotoxic T lymphocyte-mediated clearance. The up-regulation of the PD-1/ CD274 (PD-L1) axis suppressed the cytotoxic action of T cells and might reflect poor response to radiation therapy [31]. The role of PD-1/ CD274 (PD-L1), however, was not well described in response to radiation treatment. The role of CD274 (PD-L1) in the present study would not be elucidated and somehow not coincide with previous studies. CD274 (PD-L1) expression was observed in inflammatory cells in tumor stoma, but not in tumor cell in the present study. If PCRT acts like PD-1 inhibitors, CD274 (PD-L1) expressed tumor might better respond to PCRT. The mechanism how CD274 (PD-L1) worked on radiation response would be investigated in the further studies including post-PCRT tumor evaluation We did not analyze prognosis according to immune infiltrates which have been mainly studied in the previous literatures. There has been lack of studies regarding role of immune infiltrates for prediction of radio-responsiveness to PCRT in rectal cancer patients. For the purpose of evaluation of immune infiltrates as prediction markers, we need to set the category of responsiveness. We analyzed radio-responsiveness as dichotomous category. Although patients with total regression were known to show good prognosis [4, 5], residual disease in the present study include diverse spectrum of responsiveness to PCRT. Therefore the role of immune infiltrates in prognostication might not match in “dichotomous” categorized responsiveness group. Although the association of pathologic regression level and prognosis have been reported by many researchers, good prognosis in the total regression group was the most clearly defined feature [1, 4, 6]. Therefore, to find prediction marker of total regression group would be well-agreed criteria. In addition, there was lack of reports regarding association between responsiveness to PCRT and immune infiltrates in rectal cancer patients. Role of immune infiltrates in untreated rectal cancer regarding radiation responsiveness was little known. Therefore, we have to be careful to apply “theoretical” hypothesis to practical response.

We used OPAL staining and Vectra-Inform image analysis system for automated and systematized quantified evaluation of immune infiltrates. We wanted to minimize bias based on IHC and subjective pathologic review. Although IHC is one of the leading methods used to identify and co-localize antigens in cells and tissues, and has proven to be an effective and powerful diagnostic and research tool, the complex methods and protocols underlying IHC require validation and fine-tuning, detailed and complicated in design, to achieve accurate results. The complexity arises from the large diversity of cell-specific and tissue-specific molecular and macromolecular components and their modifications during sample fixation and processing. Besides, variations due to “human factor” during manual staining may lead to unreliable results. We expect that the automatized method used in the present study would reproduce stable results and confirmed by triple re-do the analysis. Indeed the automatized counting of total and specific immune infiltrated would be the base of “quantified” measurement of immune-profiling and possibility of setting cut-off point of prediction. Co-stained analysis also would be helpful to avoid double counting of unclear stained infiltrates.

The extent of “association” of specific immune infiltrates on radio-responsiveness and its correlation with other immune infiltrates have to be considered together to explore how immune background worked on radiation-therapy and how we improve radio-responsiveness.

We showed difference in correlation among specific immune infiltrates between response groups as well as certain immune infiltrates level. However, we did not perform functional evaluation of each immune-infiltrate, therefore, we would not explain how these immune infiltrates work on radiation sensitivity. We would include selected immune infiltrates and not evaluate sufficiently influence of other infiltrates. Indeed we used pre-treatment biopsy specimen. The biopsy might not be representative of whole tumor feature. However, we tried to compromise limitation of biopsy by analyzing proportion and ratio between immune infiltrates and using mean value of number of infiltrated cell. Comparing immune infiltrates before and after PCRT would be helpful to explore the mechanism associated with radio-responsiveness and we are preparing it for further study.

We found difference in specific immune infiltrates according to treatment response level to PCRT in rectal cancer. Indeed, we showed a possibility of usefulness of automatized systematized staining and counting system of immune infiltrates. We also tried to extend study in clinical setting by including CD274 (PD-L1) which was known as target for immunotherapy. Although we need to do larger scaled study as well as functional aspect of immune infiltrates in radiation therapy, we suggest the role of immune infiltrates in the prediction of radio-responsivenss to PCRT in rectal cancer patients.

## MATERIALS AND METHODS

### Patients and preoperative chemoradiotherapy

Among identified patients who underwent PCRT followed by surgical resection at Asan Medical Center between 2013 and 2015, 75 patients who were available for pretreatment biopsy tissue and posttreatment pathologic TRG were selected for this study. Pretreatment biopsy slides were reviewed by a pathologist (SMH) and one representative slide was selected for multiplexed immunofluorescence. Informed consent was waived.

Radiotherapy was given in 25 fractions to the entire pelvis, followed by a 5.4-Gy boost in 3 fractions to the primary tumor and administered dose was 45–50.4 Gy. The 5-fluorouracil, with a leucovorin, capecitabine, and oxaliplatin-based regimen was used as combination chemotherapy with radiotherapy. Two cycles of intravenous 5-fluorouracil (FU) (375 mg/m^2^/day) and leucovorin (LV) (20 mg/m^2^/day) was delivered in bolus over 3 days during the first and fifth week of RT, or oral capecitabine (1650 mg/m^2^/day) was administered twice per day during RT. At 6–10 weeks after completion of PCRT, surgical resection such as total mesorectal excision or local excision was performed.

This study was approved by the Institutional Review Board of Asan Medical Center. (Registration no:2016–1022)

### Assessment of pathologic tumor response to PCRT

Pathologic responses to PCRT were evaluated using routine hematoxylin and eosin (H&E) sections and assessed with the 5-tier TRG system [32] total regression with no residual tumor cells and only fibrotic mass; near-total regression with microscopic residual tumor (i.e., difficult to find) in the fibrotic tissue; moderate regression, dominant irradiation-related changes with residual tumor (i.e., easy to find); minimal regression, dominant tumor mass with obvious irradiation related changes; and no regression and no evidence of irradiation related changes (fibrosis, necrosis, and vascular change).

Patients were categorized into 2 tumor response groups according to primary tumor regression in TRG; total regression group (TR) *vs.* residual disease group (RD) which included near total, moderate, minimal, and no regression group.

### Multiplexed immunofluorescence (MIF) and quantification

Immunofluorescence multiplex staining was accomplished with PerkinElmer Opal kit (Perkin-Elmer, Waltham, MA). One matched Formalin-fixed paraffin-embedded (FFPE) tissue block from selected H&E pretreatment biopsy slide from each case was obtained from patients. FFPE tissues were cut in 4 μm thick sections by rotation microtome and then placed on plus charged slides. Slides were heated at least for 4 hr in a dry oven at 60°C and then rinse 100% xylene for 10 min 3 times to deparaffinize. The FFPE sections were rehydrated in a series of graded alcohols to distilled water. Antigen retrieval was performed in citrate buffer (pH 6.0) using microwave treatment (MWT). Slides were washed and blocking was performed with 3% H_2_O_2_ blocking solution followed by Dako antibody diluent. The first primary antibodies for cytokeratin (M3515, Dako, USA, dilution dilution 1:500) were incubated for 1 hour in a humidified chamber at room temperature followed by detection using the Opal™ Polymer HRP Ms+Rb kit. Visualization of Cytokeratin was accomplished using Opal 690 TSA Plus (dilution 1:50), after which the slide was placed in citrate buffer (pH 6.0) and heated using MWT. In a serial fashion, the slide was then incubated with primary antibodies for FOXP3 (ab20034, abcam, USA, dilution 1:100) for 1 hour in a humidified chamber at room temperature, followed by detection using the Opal™ Polymer HRP Ms+Rb kit. FOXP2 was then visualized using Opal 650 TSA Plus (1:50), and the slide was placed in citrate buffer (pH 6.0) for MWT. The slide was again placed in citrate buffer (pH 6.0) and subject to MWT, and then incubated with primary antibodies for CD274 (PD-L1) (E1L3N, #13684, Cell Signaling, USA, dilution 1:500) for 1 h in a humidified chamber at room temperature, followed by detection using the Opal™ Polymer HRP Ms+Rb kit. CD274 (PD-L1) was then visualized using Opal 620 TSA Plus (1:50), and the slide was placed in citrate buffer (pH 6.0) for MWT. The slide was then incubated with primary antibodies for CD4 (NCL-L-CD4-368, Leica, United Kingdom, dilution 1:200) for 1 h in a humidified chamber at room temperature, followed by detection using the Opal™ Polymer HRP Ms+Rb kit followed by visualization using Opal 570 TSA Plus (1:50). The slide was again placed in citrate buffer (pH 6.0) and heated using MWT. The slide was then incubated with the last antibody, CD8 (4B11, NB100-65729, Novusbio, USA, dilution 1:100), for 1 h in a humidified chamber at room temperature, followed by detection using the Opal™ Polymer HRP Ms+Rb kit. CD8 was visualized using Opal 520 TSA Plus (1:50). The slide was again placed in citrate buffer (pH 6.0) and heated using MWT. Nuclei were subsequently visualized with DAPI, and the section was coverslipped using HIGHDEF^®^ IHC fluoromount (ADI-950-260-0025, Enzo, USA).

### Image acquisition and quantitative image analysis

The spectral information from a multiplexed panel of targets is captured through the Vectra 3.0 Automated Quantitative Pathology Imaging System. In order for the spectral information to be reliably unmixed and quantitated, correct examples of each fluorophore emission spectra, as well as a representative autofluorescence spectrum form an unstained sample, in the context to be used, must be registered in a multispectral library. Each of the individually stained sections (Cytokeratin-Opal 690, FOXP3-Opal 650, PD-L1-Opal 620, CD4-Opal 570, CD8-Opal 520, and DAPI) was used to establish the spectral library of fluorophores required for multispectral analysis. This spectral library forms the reference of target quantitation, as the intensity of each fluorescent target is extracted from the multispectral data using linear unmixing. Immunostained sections were scanned using the Vectra 3.0 Automated Quantitative Pathology Imaging System 20 nm wavelength intervals from 420 n to 720 nm and combined these captures to create a single stack image which retained the unique spectral signature of all m-IF markers. Image files created by Vectra were analyzed using InForm 2.2.1 image analysis software (Figure 1). Each cell was identified by detecting nuclear spectral element (DAPI). The total number of FOXP3, CD274 (PD-L1), CD4, and CD8 positive cells was considered identified as the total immune cell infiltrations in the tissue. The percentage of each immune cell subset was calculated by dividing the absolute number of each subset by the total numbers of all these cells [33].

### Statistical analysis

Comparison of variables was performed with the χ2 test or Fisher exact test for qualitative variables, and the Student *t* test was used for the quantitative variables. To assess the associations between each imuune-infiltrates proportion and density, two sample *t*-test was performed and multiplicity adjustment was conducted using using Bonferroni’s method(Apha of each cells was 0.0125 ).

Probability to predict total regression of immune-infiltrates profile was evaluated using ROC (Receiver Operating Characteristic) curve. Stepwise logistic regression analysis was used to check the variables ability to predict a pathologic response. All other analyses, including evaluation of individual OR estimates, represented secondary analyses. Statistical evaluations were carried out using the statistical package SAS for Windows, Version 9.4 (Cary, NC).

## Abbreviations

PCRT: preoperative chemoradiotherapy
CD4, CD8: cluster of differentiation 4, 8
PD-L1: programmed death-ligand 1
FoxP3: forkhead box P3
TRG: tumor regression grade
TR: total regression
RD: residual disease
FFPE: formalin-fixed paraffin block
MIF: Multiplexed immunofluorescence
MWT: microwave treatment
IHC: immunohistochemistry
